# Low-Dose 68 Ga-PSMA Prostate PET/MRI Imaging Using Deep Learning Based on MRI Priors

**DOI:** 10.3389/fonc.2021.818329

**Published:** 2022-01-26

**Authors:** Fuquan Deng, Xiaoyuan Li, Fengjiao Yang, Hongwei Sun, Jianmin Yuan, Qiang He, Weifeng Xu, Yongfeng Yang, Dong Liang, Xin Liu, Greta S. P. Mok, Hairong Zheng, Zhanli Hu

**Affiliations:** ^1^ Lauterbur Research Center for Biomedical Imaging, Shenzhen Institute of Advanced Technology, Chinese Academy of Sciences, Shenzhen, China; ^2^ Computer Department, North China Electric Power University, Baoding, China; ^3^ Chinese Academy of Sciences Key Laboratory of Health Informatics, Shenzhen, China; ^4^ Department of Nuclear Medicine, Nanjing First Hospital, Nanjing Medical University, Nanjing, China; ^5^ United Imaging Research Institute of Intelligent Imaging, Beijing, China; ^6^ Central Research Institute, United Imaging Healthcare Group, Shanghai, China; ^7^ Biomedical Imaging Laboratory (BIG), Department of Electrical and Computer Engineering, Faculty of Science and Technology, University of Macau, Avenida da Universidade, Macau SAR, China

**Keywords:** PET/MRI, prostate, low-dose restoration, deep learning, discrete wavelet transform

## Abstract

**Background:**

68 Ga-prostate-specific membrane antigen (PSMA) PET/MRI has become an effective imaging method for prostate cancer. The purpose of this study was to use deep learning methods to perform low-dose image restoration on PSMA PET/MRI and to evaluate the effect of synthesis on the images and the medical diagnosis of patients at risk of prostate cancer.

**Methods:**

We reviewed the 68 Ga-PSMA PET/MRI data of 41 patients. The low-dose PET (LDPET) images of these patients were restored to full-dose PET (FDPET) images through a deep learning method based on MRI priors. The synthesized images were evaluated according to quantitative scores from nuclear medicine doctors and multiple imaging indicators, such as peak-signal noise ratio (PSNR), structural similarity (SSIM), normalization mean square error (NMSE), and relative contrast-to-noise ratio (RCNR).

**Results:**

The clinical quantitative scores of the FDPET images synthesized from 25%- and 50%-dose images based on MRI priors were 3.84±0.36 and 4.03±0.17, respectively, which were higher than the scores of the target images. Correspondingly, the PSNR, SSIM, NMSE, and RCNR values of the FDPET images synthesized from 50%-dose PET images based on MRI priors were 39.88±3.83, 0.896±0.092, 0.012±0.007, and 0.996±0.080, respectively.

**Conclusion:**

According to a combination of quantitative scores from nuclear medicine doctors and evaluations with multiple image indicators, the synthesis of FDPET images based on MRI priors using and 50%-dose PET images did not affect the clinical diagnosis of prostate cancer. Prostate cancer patients can undergo 68 Ga-PSMA prostate PET/MRI scans with radiation doses reduced by up to 50% through the use of deep learning methods to synthesize FDPET images.

## Introduction

Prostate cancer is one of the most common cancers worldwide, with approximately 1.41 million new cases reported in 2020, the third most common among 36 cancers (1). In recent years, studies have demonstrated that 68 Ga-prostate-specific membrane antigen (PSMA) PET/MRI provides accurate staging of primary prostate cancer with a high detection rate (2–6). In terms of evaluating recurrent prostate cancer, this imaging technique also has a high detection rate, even for patients with extremely low levels of prostate specific antigen (PSA; <0.5 ng/ml). Additionally, it plays an important role in tumor detection, preliminary staging, treatment response assessment, and treatment planning (7, 8).

However, this technique also has some limitations, including scanning time, the cost of the associated radiopharmaceuticals, and the radiation delivered by PET imaging (9). The economic factors and radiation risks have different kinds of impact on the patients. The purpose of reducing the dose of radiopharmaceuticals is related to the potential risks of ionizing radiation. To reduce the risk of radiation exposure that those involved in the scan may face, especially pediatric patients and volunteers, or when a variety of different tracers are used for follow-up or to monitor treatment response, fewer radiopharmaceuticals should be used to perform PET imaging. The reduction in the number of radiopharmaceuticals will reduce the quality of the PET images, thereby affecting quantitative analysis and clinical diagnosis.

In recent years, deep learning has entered various fields of medical imaging. Hu Chen et al. used learned experts’ assessment-based reconstruction network to reconstruct CT directly from sinogram data (10). Maosong Ran et al. proposed a Parallel Dual-Domain Convolutional Neural Network for Compressed Sensing MRI to deal with the k-space and spatial data simultaneously (11) and a parameter-dependent framework to process the CT data with different scanning geometries and dose level in a unified network (12). Wenjun Xia et al. simultaneously leverage the spatial convolution to extract the local pixel-level features from the images and incorporate the graph convolution to analyze the nonlocal topological features in manifold space for low-dose CT reconstruction (13). Chenyu Shen et al. leveraged deep regularization by denoising from a Bayesian perspective to reconstruct PET images from a single corrupted sinogram without any supervised or auxiliary information (14). Researchers have proposed a variety of methods to ensure that the synthesized FDPET images have the same image quality as the clinical diagnostic images (15–17). In particular, deep learning has shown great potential in recovering LDPET images. Yan Wang et al. used a 3D conditional generative adversarial network (GAN) to synthesize FDPET images from head LDPET images (18). Wenzhuo Lu et al. used a fully optimized 3D U-net to effectively reduce the noise in LDPET images from the lungs while minimizing the deviation in the lung nodules (19). Yang Lei et al. proposed using CycleGAN to estimate prostate FDPET images from prostate LDPET images (20). Long Zhou et al. also used CycleGAN, denoising low-dose fluorodeoxyglucose (FDG) PET images and subsequently performing quantitative analysis on the images (21).

Deep learning also has a variety of exploratory research in prostate PET images. Pablo Borrelli et al. used convolutional neural network to detect lymph node metastases by PET/CT predicting prostate cancer-specific survival (22). Eirini Polymeri et al. evaluated a novel three-dimensional deep learning-based technique on PET/CT images for automated assessment of cancer in the prostate gland and its agreement with manual assessment (23). Sangwon Han et al. evaluated the performance of deep learning classifers for bone scans of prostate cancer patients (24). Dejan Kostyszyn et al. examined the capabilities of convolutional neural network for intraprostatic GTV contouring in 68Ga- and 18F-PSMA-PET (25). Sobhan Moazem et al. used UNet to predict treatment response in prostate cancer patients based on multimodal PET/CT for clinical decision support (26). Andrii Pozaruk et al. developed a novel augmented deep learning method based on GANs for accurate attenuation correction in the simultaneous PET/MR scanner (27).

In this study, we retrospectively analyzed the 68 Ga-PSMA PET/MRI data of 41 patients in Nanjing First Hospital of China. The PET images were reconstructed at acquisition times of 2.5%, 5%, 25%, 50%, and 100% of the standard acquisition time. A discrete-wavelet-transform convolutional neural network (DWTN) was used to restore the LDPET images to the original, FDPET images with or without the use of MRI priors, respectively, to explore the extent to which this method can reduce the required radiotracer dose.

## Materials and Methods

### PET/MRI Data Acquisition

In this study, we used clinical images obtained from 68 Ga-PSMA PET/MRI examinations performed at Nanjing First Hospital of China from January 2021 to July 2021. The data were obtained from 41 male patients who might present with signs of future prostate cancer. The mean age of the patients was 67 ± 6 years, and the mean weight was 73 ± 10 kg. The research protocol was approved by the institutional ethics committee, and all patients were provided written informed content. Sixty minutes after the patient had been injected with 68 Ga-PSMA (in the range of 111-185×10^6^ MBq), scanning data were collected from the PET/MRI scanner (United Imaging Healthcare, uPMR 790). The acquisition time of emission images was 600 seconds, and the PET images were reconstructed at acquisition times of 600, 300, 150, 30, and 15 seconds.

All PET images were reconstructed using ordered subset expectation maximization (OSEM) algorithm and a set series of parameters, for example, 3D iterative time-of-flight (TOF) and point-spread function (PSF) reconstruction, 2 iterations, 20 subsets, matrix 192×192, slice thickness 2.5 mm, and correction methods such as decay correction, attenuation correction, scatter correction, dead time correction, random correction, and detector normalization correction. The images reconstructed at the acquisition times above correspond to 100%-, 50%-, 25%-, 5%-, and 2.5%-dose (low-dose) PET images, respectively. The 100% does PET images were used as ground-truth and the remaining dose PET images were used as input images. The water sequence decomposed from the T1-weighted MRI images was used as the prior images for generating the LDPET images.

Since integrated prostate PET/MRI was used for scanning, the PET and MRI scans were coaxial. The PET image matrix size is 192×192, and the MRI image matrix size is 552×387. To ensure that the image resolution was not lower than that of the original image, we used bicubic interpolation to resize the two modal images as a 512×512 matrix. Since the image matrix sizes of the two modalities are now the same, the images of the two modalities do not need to be registered. In total, the 41 patients had 4100 sets of images. These sets are divided into training data set and test data set. We used 90% of the data set to train the model and the remaining 10% to verify the effect of the images generated by the model. To avoid overfitting due to the small size of the training data set, we increased the number of images in the training data set by flipping the images down, left and right, quadrupling the size of the training data set. This process helped improve the generalizability of the deep learning models.

### Discrete-Wavelet-Transform Convolutional Neural Network

**Figure 1:**
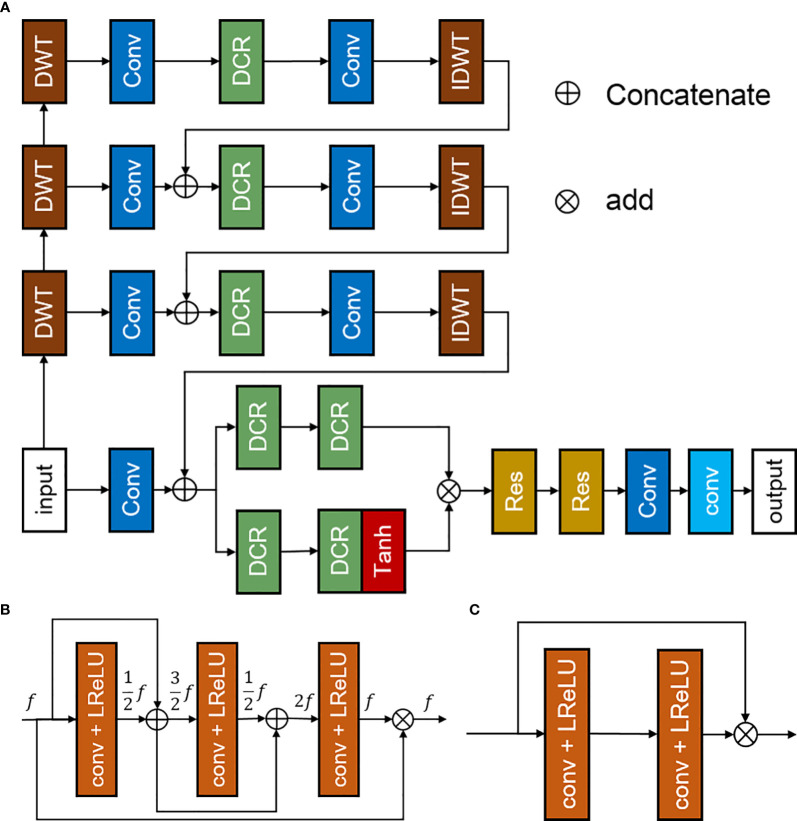
Discrete-wavelet-transform neural network (DWTN), including **(A)** the structure of the DWTN network, **(B)** the structure of the densely connected residual (DCR) block, and **(C)** the structure of the residual block.

The discrete-wavelet-transform convolutional neural network (DWTN) proposed in this study is an improvement of the densely self-guided wavelet network (28), which is suitable for LDPET image restoration tasks based on MRI priors. The structure of DWTN was shown in **Figure 1**. The multilayer self-guided architecture makes better use of multiscale image information; low-resolution feature information from the top layer is gradually fused with higher-resolution feature information to improve the network’s ability to extract multiscale feature information from images. Wavelet transform is used instead of ordinary upsampling and downsampling and PixelShuffle and PixelUnshuffle to generate multiscale image information. Before the convolution process, the image is converted into horizontal, vertical, and diagonal detail images and thumbnails through discrete wavelet transform (29). At the full-resolution layer, the main branch and attention branch provide stability and process the feature images. At each layer, we add densely connected residual blocks to improve the convergence of the network. The top layer of the DWTN extracts large-scale image feature information in the lowest resolution space. The top-level network contains two convolution layers, a leaky ReLU layer, and a densely connected residual (DCR) block (30). As shown in **Figure 1** (B), the DCR block consists of three convolutional layers, each of which is followed by a leaky ReLU. Each feature image is connected by dense connections so that our model can use the previous feature information to solve the gradient disappearance problem. The middle two layers are similar to the top layer. For the full-resolution level, we add multiple DCR blocks after merging the multiscale feature information to enhance the feature extraction capability of the DWTN. For the attention branch, we add a tanh activation function after the two DCR blocks. The main branch and the attention branch are processed and added, and then through multiple residual blocks and convolution blocks, the added image feature information is extracted. The structure of residual block was shown in **Figure 1** (C). Finally, through a convolution block without an activation function, the details of the image are preserved. The last convolution layer uses a 1×1 convolution kernel with a step count of 1, and the remaining convolution layers all use a 3×3 convolution kernel with a step count of 1.

To improve the generalization of the network, we used perceptual loss, mean-square-error loss and kernel loss to constrain the network (31). Among them, perceptual loss enabled the network to learn the characteristics of the overall images and converge faster than mean-absolute-error loss. The perceptual loss used pre-trained VGG19 for extracting image features. The mean-square-error loss was to calculate the loss function at the pixel level to ensure the network to generate more details of the images. The kernel loss reduced the weight of the hidden layer inside network, thereby promoting the convergence of DWTN. The loss function formula was as follows:


(1) $${\text{Loss}}_{total}=mse\times {\text{Loss}}_{MSE}+vgg\times {\text{Loss}}_{per}+kl\times {\text{Loss}}_{ker},$$

where mse, vgg and kl are 0.5, 0.5 and 0.0001, respectively. The formulas of each loss function are as follows:


(2) $${\text{Loss}}_{MSE}=E{\left(\hat{y}-y\right)}^{2},$$

(3) $${\text{Loss}}_{per}=E{(\text{VGG}(\hat{y})-\text{VGG(y))}}^{2},$$

(4) $${\text{Loss}}_{ker}=E{\left(\hat{y}\right)}^{2},$$

where $\hat{y}$ and *y* represent the generated images and ground-truth, respectively. VGG represents the processing of VGG model.

When initializing the training parameters, we used the ADAM optimizer, and the remaining parameters are set to their default values. We update the learning rate every 25 epochs and set the learning rate decay rate to 0.5. The weights of all hidden layers are initialized with Gaussian random numbers. The model was implemented on an NVIDIA GeForce RTX 2080Ti GPU with 11 GB of memory and run under the Microsoft Windows 10 operating system. During training, we used a batch size of 4 for 100 epochs.

### Evaluation Metrics

Clinical quantitative evaluation. To evaluate the quality of the PET images, we evaluated the original LDPET images, the synthesized FDPET images, and the synthesized FDPET images based on the MRI priors. There were 13 sets of images, including 32 PET images in each group. The PET images were evaluated using a 5-point method by two nuclear medicine physicians from Nanjing First Hospital of China (32, 33).

Image quantitative analysis. To evaluate the image quality between the synthesized FDPET images and the original FDPET images, we used the PSNR, SSIM, RCNR, and NMSE as objective indicators.

The PSNR is a quantitative index for evaluating images and noise, and the SSIM is an index for evaluating the similarity of two image features; both indices offer comprehensive evaluations of two images.


(5) $$\text{PSNR}=10\times \log_{10}\left(\frac{{\left(2^{n}-1\right)}^{2}}{MSE}\right),$$

Where MSE is the mean square error between the compared images and the ground-truth.


(6) $$\text{SSIM}=\frac{\left(2\mu_{x}\mu_{y}+c_{1})(2\sigma_{xy}+c_{2}\right)}{\left(\mu_{x}^{2}+\mu_{y}^{2}+c_{1})(\sigma_{x}^{2}+\sigma_{y}^{2}+c_{2}\right)}$$

where *μ_x_* is the mean of compared images and *μ_y_* is the mean of ground-truth, respectively. *σ_x_* and *σ_y_* are variance of compared images and ground-truth, respectively. *σ_xy_* is the covariance between compared images and ground-truth. *c*_1_ and *c*_2_ are 0.01 and 0.03, respectively.

The NMSE is an indicator of the quantitative analysis of two images at the pixel level.


(7) $$\text{NMSE}=\frac{\Sigma_{i\in V}{\left(X_{i}-Y_{i}\right)}^{2}}{\Sigma_{i\in V}{\left(Y_{i}\right)}^{2}},$$

Where *X_i_* and *Y_i_* represent the pixel value of the compared image and groud-truth, respectively.

The contrast-to-noise ratio is an objective index used to evaluate the quality of medical images; the RCNR is a dimensionless image index based on CNR that is used to compare the contrast of two images.


(8) $$\text{CNR}=\frac{X-X_{background}}{X}$$

(9) $$\text{RCNR}=\frac{CNR_{x}}{CNR_{y}},$$

Where X and *X_background_* represent the mean of the matrix and background matrix, respectively.

## Results

Compared with the LDPET images, the FDPET images synthesized by the deep learning method demonstrated significantly improved image quality. PET images with a dose of less than 5% showed irregular spots, and their contours, shapes, and contrast were different from those of the target images. In the images synthesized from those with a dose of less than 5%, the spots were eliminated, and the shape features and contrast were relatively consistent with those of the target images. In PET images with a dose greater than 5%, the contours, shapes, and contrast were similar to those the target images, but subtle differences could be observed. In the synthesized PET images, the shapes, contours, and contrast were consistent with those of the target images.

**Figure 2:**
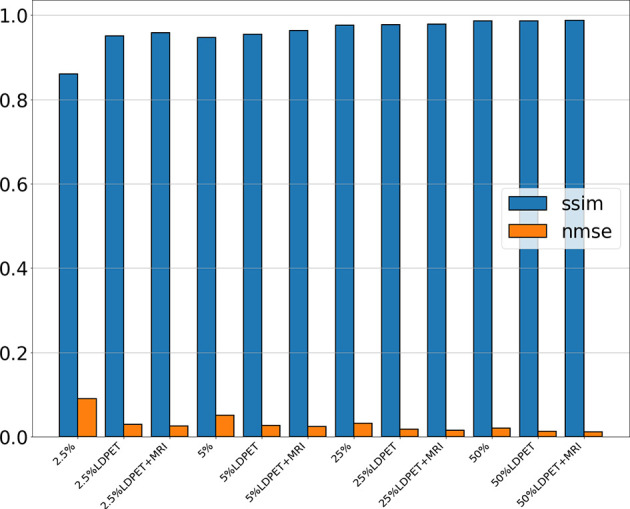
NMSE and SSIM of the original LDPET images, synthetic FDPET images, and MR prior-synthesized FDPET images at all doses, where blue represents SSIM, orange represents NMSE, and different degrees of color represent images of different doses.

After training the model, we calculated the mean and standard deviation of the RCNR, PSNR, SSIM, NMSE among the original LDPET original image, the synthesized FDPET images, the prior-synthesized FDPET images, and the target images of all doses in the test set. **Table 1** shows the mean and standard deviation values of the image indicators. To more intuitively visualize the differences in these image indicators, **Figure 2** shows the histograms of the structural similarity and NMSE indicators. The processed LDPET images had significantly better image quality than the original LDPET images. The images based on MRI prior synthesis showed better image quality at the global-feature and pixel levels.

**Table 1:** Objective indicators for LDPET images, synthesized FDPET images, MR prior-synthesized FDPET images, and target images.

Image	PSNR	SSIM	NMSE	RCNR
2.5%	25.56±4.99	0.745±0.112	0.065±0.045	0.835±0.267
2.5%LDPET	32.51±4.89	0.820±0.079	0.029±0.021	1.046±0.213
2.5%LDPET+MRI	33.34±4.47	0.846±0.060	0.026±0.018	1.118±0.218
5%	26.99±5.56	0.756±0.113	0.057±0.037	0.860±0.227
5%LDPET	33.25±4.57	0.814±0.137	0.026±0.018	1.032±0.216
5%LDPET+MRI	33.78±4.25	0.817±0.141	0.024±0.017	0.964±0.248
25%	29.58±7.05	0.832±0.114	0.047±0.036	0.901±0.195
25%LDPET	36.90±4.40	0.893±0.090	0.017±0.012	1.035±0.123
25%LDPET+MRI	37.86±4.16	0.916±0.063	0.015±0.012	1.004±0.126
50%	32.02±8.66	0.865±0.123	0.040±0.036	0.909±0.183
50%LDPET	39.48±3.90	**0.919**±**0.067**	0.012±0.008	1.009±0.079
50%LDPET+MRI	**39.88**±**3.83**	0.896±0.092	**0.012**±**0.007**	**0.996**±**0.080**

In bold: The best performance in this indicator.

**Figure 3:**
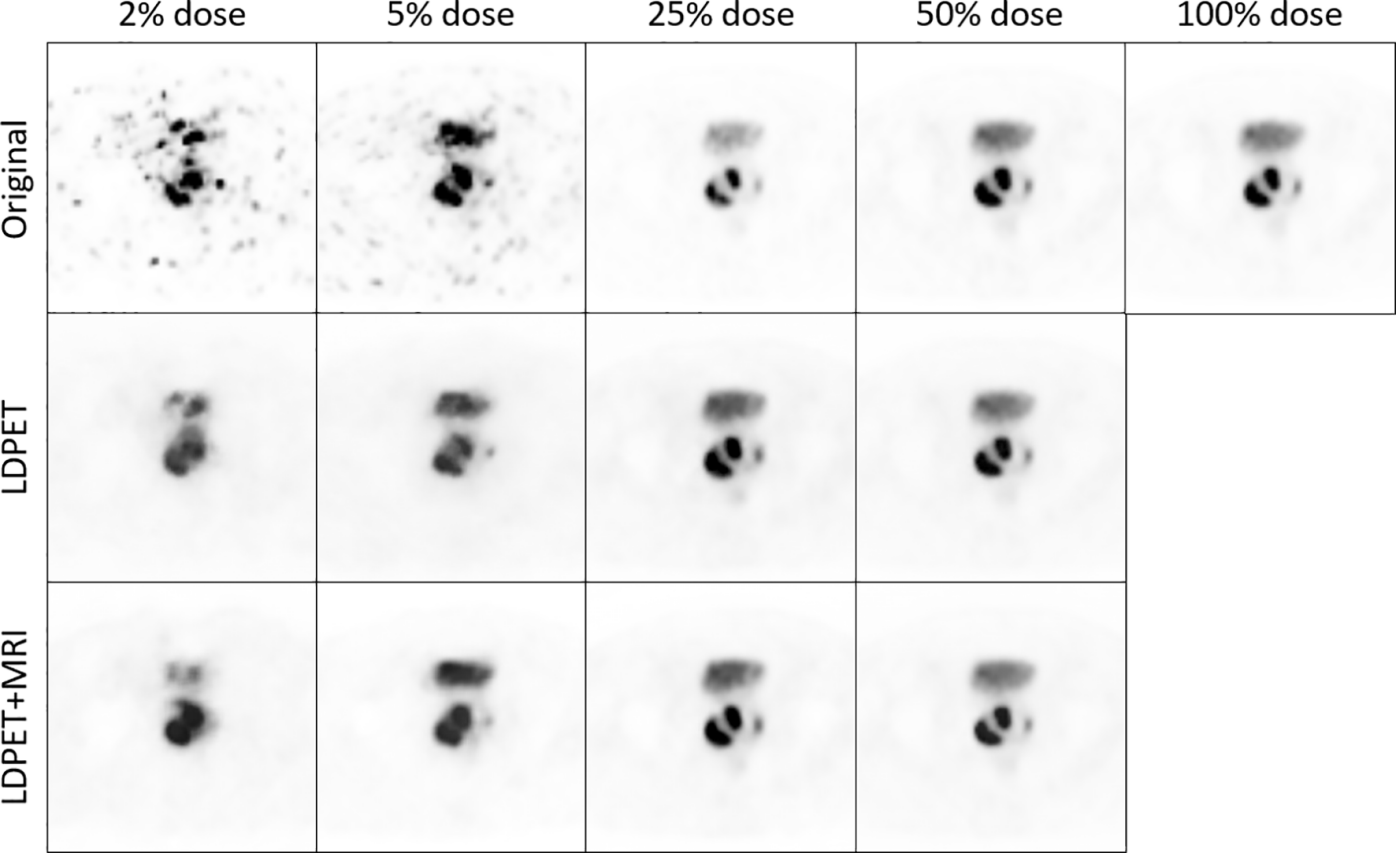
The original LDPET images, synthesized FDPET images, MR prior-synthesized FDPET images of all doses, and their ROIs.

**Figure 3** shows one patient’s original 2.5%-, 5%-, 25%-, and 50%-dose PET images and their corresponding synthesized FDPET images and prior-synthesized FDPET images for the prostate. The average RCNR of the LDPET images and MRI prior-based synthesized LDPET images was close to 1. Moreover, the pelvic contour details of the prior-based synthesized images for doses of 25% and above are visible.

**Figure 4:**
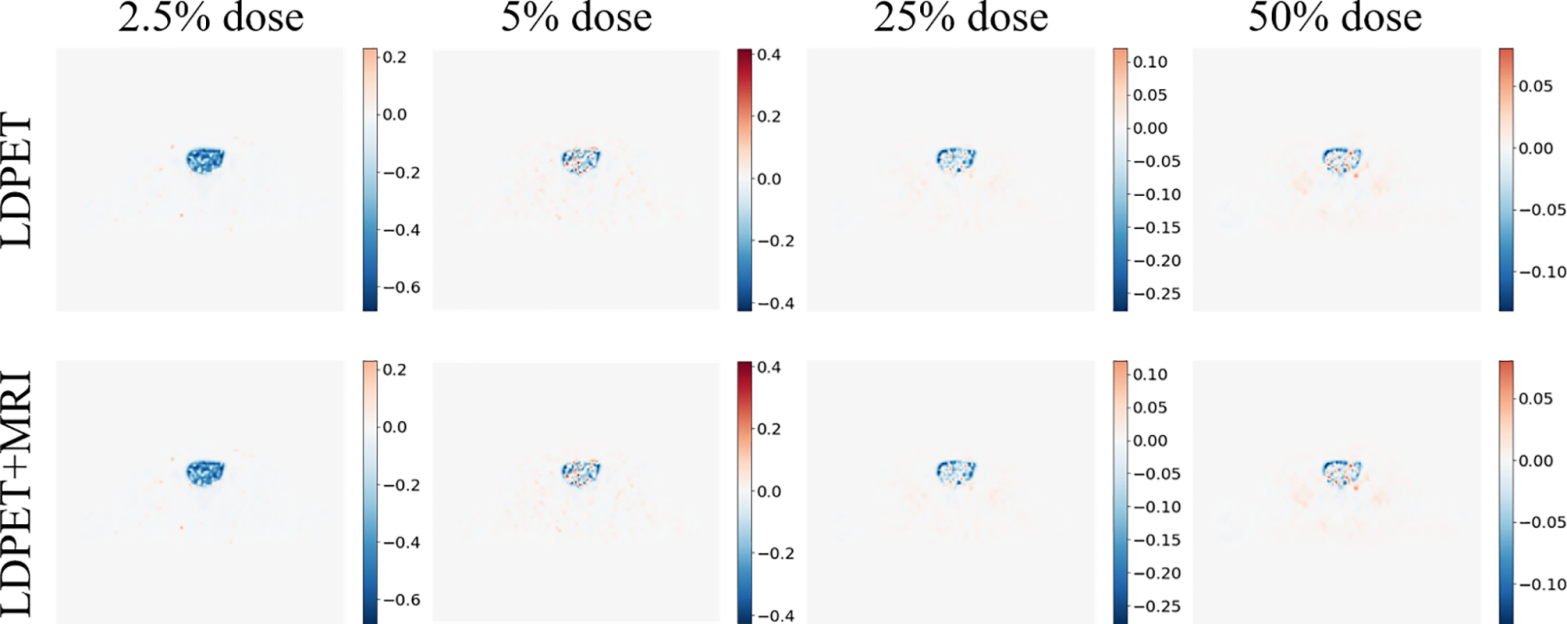
Synthesized FDPET image, MR prior for all doses, combined FDPET image and target subtraction difference map and original image.

We transferred the synthesized images and MRI prior synthesized images for each LDPET image to DICOM format, subtracted each image from the original FDPET image matrix, and finally divided the difference matrix by the maximum value of the original image. The resulting image matrixes are shown in **Figure 4**. The error between the 25%-dose synthesized images and the prior-synthesized images are within 25%, and the error between the 50%-dose synthesized images and the prior-synthesized images are within 10%.

**Figure 5:**
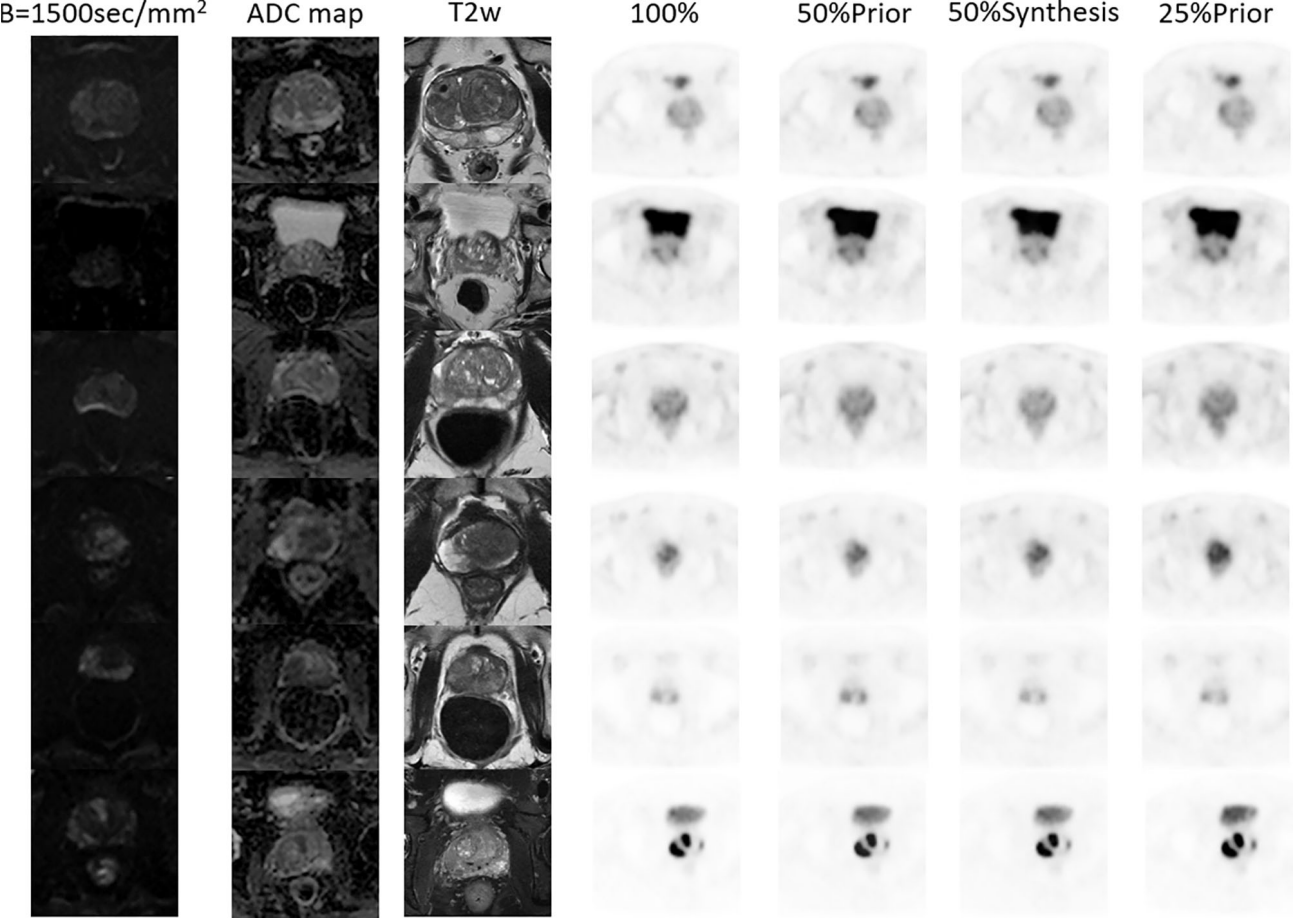
MR and PET images of 6 patients with prostate or pelvic lesions from the test set. The MR sequences included DW-, ADC- and T2-weighted images. The PET images consist of the original FDPET images, FDPET images synthesized from 50%-dose images with MR priors, FDPET images synthesized from 50%-dose images without MR priors, and FDPET images synthesized from 25%-dose images with MR priors.

Six patients were selected from the test set, all of whom had images of prostate or pelvic lesions, as shown in **Figure 5**. The figure shows the diffusion weighted (DW) image, apparent diffusion coefficient (ADC) image, T2-weighted image, FDPET image, and various synthesized images. The DW image, ADC image, and T2-weighted image in the MRI sequence are important references for clinical diagnosis, and the PET images and MRI images are complementary.

**Figure 6:**
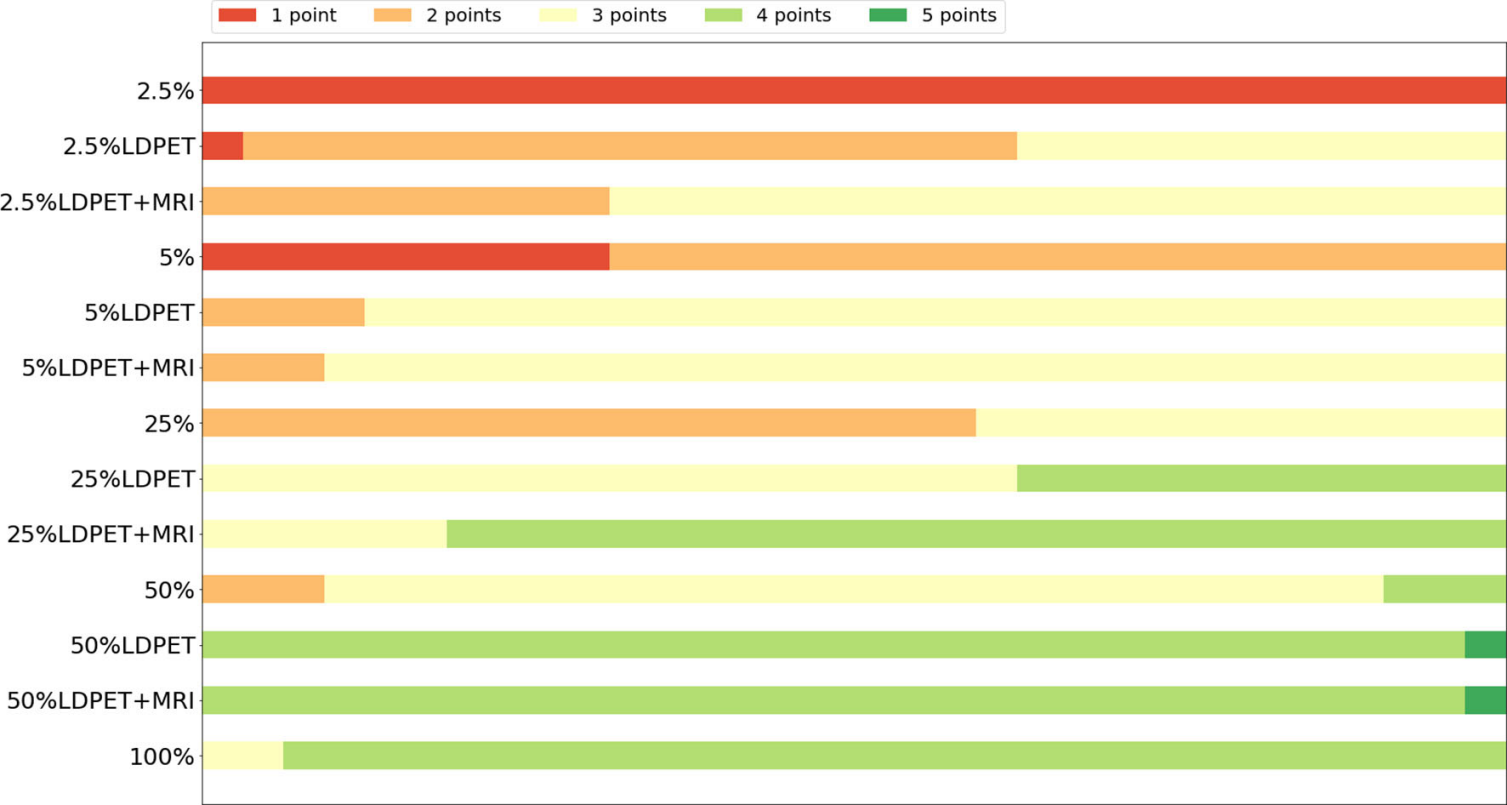
Distribution of clinical quantitative scores from nuclear medicine doctors on LDPET images, synthesized FDPET images, MR prior-synthesized FDPET images, and target images.

In the clinical quantification phase, we selected 32 images with lesions in the pelvis or prostate from the test set for scoring. **Table 2** shows the mean and variance of the scored from the two nuclear medicine doctors for PET images of different doses, processed PET images, and prior-based PET images. The average score of the FDPET images MRI prior-synthesized from 25%-dose PET images is 0.1 points lower than the average score of the target images. In contrast, the average score of the FDPET images synthesized from 25% dose PET images without the prior is 0.3 points lower than the average score of the target images. In addition, to improve the credibility of the analysis, we combined the scores of the two doctors. When the two nuclear medicine doctors had different scores for the same image, then the lower score of the two was taken. **Figure 6** shows the distribution of this score. When MRI images were used as the prior, more than 80% of the FDPET images synthesized from 25%-dose PET images had scores of 4, while the rest had scores of 3. When no priors were used, the scores of the FDPET images synthesized from the 25%-dose images indicated that the FDPET images were between good and poor quality. Regardless of whether MRI images were used as priors, the scores of the FDPET image synthesized from the 50%-dose PET images were greater than or equal to 4. At each dose, using MRI images as priors in synthesizing the images was better than using single LDPET images to synthesize FDPET images.

**Table 2:** Mean clinical quantitative scores from nuclear medicine doctors on LDPET images, synthesized FDPET images, MR prior-synthesized FDPET images, and target images.

	Original	LDPET	LDPET+MRI
2.5%	1.00±0.00	2.44±0.56	2.69±0.46
5%	1.69±0.46	2.94±0.43	3.28±0.57
25%	2.47±0.50	3.62±0.48	3.84±0.36
50%	3.03±0.39	4.03±0.17	4.03±0.17
100%	3.94±0.24	–	–

## Discussion

In the LDPET image denoising method, MRI images were used as prior image to provide rich tissue and anatomical information for PET image synthesis, thereby improving image quality, contours and details. However, the use of MRI as priors can also lead to registration problems. Due to the characteristics of the MRI images and the PSMA PET images, accurate registration, regardless of whether rigid registration or flexible registration is used, can be difficult. Data registration is not a problem, however, because the integrated PET/MRI device uses coaxial scanning, and the data it collects can be directly applied to LDPET restoration. Theoretically, data collected by integrated PET/MRI are more suitable for MRI prior-based LDPET estimation than data collected by sequential PET/MRI, insert PET/MRI, or multiple devices.

When using a single LDPET image for denoising, synthesized prostate FDPET images have poor overall contour and edge details and cannot be restored well. When including MRI images as priors for LDPET image denoising, the edges of PET images with a dose of less than 5% is blurred, and the surrounding contours are not clear, which can affect the diagnosis. In PET images with a dose higher than 5%, the shape and edges of the key parts of the prostate are clear, the contours of the surrounding organs are distinct, the contrast is relatively close to that of the target image, and the clinical quantitative score is the same as that of the target image. However, **Figure 3** shows that the 25%-dose PET synthesized images based on the MRI priors and the ground-truth have a large error in the local area of the prostate. Thus, the 50%-dose PET images synthesized based on the MRI priors showed sufficient quality to meet the requirements for clinical analysis.

However, our method still has certain limitations. First, the proposed network uses the Haar wavelet transform for up- and downsampling. Other potential wavelet transforms include Gaussian, Morlet, Shanno, and other transformation methods, and whether the Haar wavelet transform is the most suitable for MRI prior-based LDPET estimation is not yet known. Second, the proposed network has a significant effect on LDPET images with good overall characteristics but has a poor effect on LDPET images with inconspicuous overall characteristics. The recovery effect of the convolutional neural network on PET images with a dose of 5% and below needs to be improved; for example, the use of generative adversarial neural networks for image recovery and PET/MRI examinations could reduce the dose even further.

## Conclusion

In conclusion, we used a convolutional neural network combining discrete wavelet transform and convolution methods to estimate FDPET images from PSMA LDPET image collected by PET/MRI. After clinical quantitative analysis and objective image index analysis, the deep learning method we proposed was shown to be capable of synthesizing a FDPET image from 50%-dose PSMA PET images collected by PET/MRI, indicating that the dose can be reduced by 50%.

## Data Availability Statement

Further inquiries can be directed to the corresponding author. Requests to access the datasets should be directed to Zhanli Hu, zl.hu@siat.ac.cn.

## Ethics Statement

The studies involving human participants were reviewed and approved by (Permit S-152/2020) Department of Nuclear Medicine, Nanjing First Hospital, Nanjing Medical University. The patients/participants provided their written informed consent to participate in this study.

## Author Contributions

FD was responsible for programming and revising the manuscript. X-yL and FY performed data acquisition and analyzed the data. HS, JY, QH, YY, DL, XL, GM, HZ and WX made a substantial contribution to the study and helped revise the manuscript critically for important intellectual content. ZH made substantial contributions to the conception and design of the study. All authors read and approved the final manuscript.

## Funding

This work was supported by the National Natural Science Foundation of China (32022042, 81871441), the Shenzhen Excellent Technological Innovation Talent Training Project of China (RCJC20200714114436080), the Natural Science Foundation of Guangdong Province in China (2020A1515010733), Chinese Academy of Sciences Key Laboratory of Health Informatics in China (2011DP173015).

## Conflict of Interest

HS, JY, and QH are employees of the United Imaging Healthcare group.

The remaining authors declare that the research was conducted in the absence of any commercial or financial relationships that could be construed as a potential conflict of interest.

## Publisher’s Note

All claims expressed in this article are solely those of the authors and do not necessarily represent those of their affiliated organizations, or those of the publisher, the editors and the reviewers. Any product that may be evaluated in this article, or claim that may be made by its manufacturer, is not guaranteed or endorsed by the publisher.
